# A Mathematical Model of Estradiol Production from Ultrasound Data for Bovine Ovarian Follicles

**DOI:** 10.3390/cells11233908

**Published:** 2022-12-02

**Authors:** Malgorzata J. McEvoy, Marion McAfee, John A. Hession, Leo Creedon

**Affiliations:** Centre for Mathematical Modelling and Intelligent Systems for Health and Environment (MISHE), Atlantic Technological University, Ash Lane, F91 YW50 Sligo, Ireland

**Keywords:** estradiol, ovarian follicles, atresia, ultrasound

## Abstract

In this paper, we present a new way to assess the concentration of estradiol (E2) and Insulin Growth Factor-1 (IGF) based on the results from ultrasound scans combined with mathematical models. The IGF1 model is based on the progesterone (P4) concentration, which can be estimated with models calculating P4 level based on the size/volume of corpus luteum (CL) measured during ultrasound scans. At this moment little is known about the underlying reasons for double ovulation and silent heat occurrences. Both of these are linked to the level of IGF1: double ovulations are linked to higher IGF1 levels and and silent heat is linked to lower E2 to P4 ratio. These models can help to improve understanding of the related concentrations of E2 and IGF1. Currently, it is known that diet and genetic factors have an impact on ovulation rates and silent heat. In this study, we also examine the decline of the production of E2 in vivo by atretic follicles throughout the process of atresia. This is the first recorded quantitative description of this decline.

## 1. Introduction

E2 production dynamics are important in bovine (and other mammalian) reproductive cycles because the estradiol (E2) produced by ovarian follicles plays an important role in governing the bovine estrous cycle and has a direct effect on the reproductive capability of the animals. Profitability of grass fed cattle is based on a 365 day reproduction cycle, which means that a cow has to have offspring and become pregnant again within 3 months. This minimizes the nutrition expenses and maximises output of milk and beef. E2 level has an impact on the expression of estrus (elevated level of estrogen and low concentration of progesterone (P4)). The concentration of hormones (higher P4 and lower E2) can prevent/reduce estrus signs and result in `silent heat’ [1]. Silent heat results in greater expense, lower production and more culling of healthy, potentially productive cows. In bovines, there is a surge of estradiol 10–11 h prior to ovulation (coinciding with low levels of P4) [2]. Thus, it is important to understand changes in the production of estradiol throughout the lifespan of the ovarian follicle.

The “fast” phase of follicular growth lasts approximately 5–7 days and is gonadotrophin dependent. It consists of cohort growth and dominant follicle selection (generally only 1 follicle is allowed to grow). This phase is dependent on FSH and LH concentrations. Development of follicles from 4 mm always requires an increase in FSH from baseline levels [3]. Research conducted by Gutierrez et al. (1997) [4] shows that follicles can grow to 4 mm without FSH; thereafter FSH was needed for follicle growth [5]. A concentration of estradiol 2–3 times higher than normal estradiol levels during the follicular phase can suppress the growth of the follicles [3]. After recruitment, 3 to 6 follicles with a diameter of 4 to 5 mm constitute a follicular wave [6]. Small emerging follicles produce very small amounts of estradiol and inhibin per follicle but taken together those quantities are not negligible [7]. After 2 days of follicle recruitment (usually) one follicle is selected and the others undergo atresia [8]. The deviation denotes the beginning of the distinctive growth rate difference between follicles [9,10]. The time of deviation can be assessed by observing the diameters of the two largest follicles. The dominant follicle emerges approximately 6 h before any future subordinate follicle [11]. Follicle deviation takes place approximately when the two largest follicles reach 8.5 and 7.7 mm, respectively [11]. There are physiological changes between these two follicles regarding local (e.g., IGF and IGF binding proteins) and systemic mechanism regulation (e.g., FSH concentrations) [11,12].

The dominance of a follicle can be observed by its diameter, using ultrasonography or rectal palpation. Dominant follicles have been defined to be ≥10 mm in diameter [11,13]. There can be multiple dominant follicles in a wave; either they are induced by gonadotropin treatment or are spontaneous. The maximum diameter reaches approximately 11 mm in *Bos indicus* [14] and 16 mm in *Bos taurus* [15]. The dominance is associated with physiological (e.g., granulosa cell LH receptors, vascularity) and endocrinological (follicular fluid inhibin and estradiol concentration) aspects of the follicle during its growth. The dominant follicle indirectly causes regression of subordinate follicles and suppression of new wave emergence through suppression of FSH surge. The FSH suppression is associated with change in LH binding and activation of enzymes producing estradiol [7]. Selected follicles develop LH receptors at 6.8 mm [16], which allows the dominant follicles to grow in a LH environment at low FSH levels [17]. The follicular wave dynamics is repeated throughout the estrus cycle and also during pregnancy [13].

FSH and LH are necessary for promotion of follicles (FSH for follicles < 8 mm in diameter and LH for follicles > 8 mm in diameter). These hormones are not modelled directly (to simplify possible future use of a model in field conditions), but sizes of follicles less than and greater than 8 mm are distinguished. Furthermore, data for LH and FSH was unavailable. Instead, IGF1 data was used indirectly as a proxy for follicle (and estradiol) promotion. If LH and FSH data are available, then they could also be used [18].

There is an elevation in LH level before deviation, followed by LH decrease just after deviation [9,19]. For dominant follicles to grow greater than 8 or 9 mm requires LH [9,20] and they can reach 12–20 mm (this takes 3–4 days) [21]. One day after the start of deviation, follicles acquired ovulatory capacity (at 10 mm diameter), and they need a surge of LH to ovulate. The surge of LH can only take place in a low progesterone (P4) environment, due to P4 influence on LH pulsatility [21]. At high progesterone level, the dominant follicle (DF) undergoes atresia and its functional dominance is lost between day 7 and day 9 of the estrous cycle, preceded by a decline in estradiol secretion (around day 6) by the DF of the first follicular wave [21].

During the estrus cycle there are on average 1 to 2 non-ovulatory waves and one ovulatory wave. The ovulatory wave is the wave which results in a follicle ovulating. A follicle is called a preovulatory dominant follicle if it is a dominant follicle in the ovulatory wave (and typically it ovulates). The number of follicular waves can vary between 1 and 4, and affects the cycle length [17]. The duration of the estrous cycle varies; those lasting on average 20 [7] or 21 [17] days usually come with two follicular waves; those of 22 [7] or 23 [17] days usually have three follicular waves [17]. Luteolysis of CL allows for final maturation of the dominant follicle, followed by ovulation and the formation of new CL [22] or pregnancy. The follicular dynamics was recorded for development of follicles from approximately 5 mm in diameter to the ovulatory size. The ultrasonography allows the follow-up of growing follicles (≥2 mm) on a daily basis [17].

The majority of the current mathematical models of E2 production do not relate E2 to the number and sizes of the follicles present at a given time [7,23,24,25]. Boer et al. (2012) introduced the function ’Foll’, which stands for the summed diameters of the follicles present at a given time, however it does not differentiate between growing and atretic follicles. Furthermore, the E2 levels calculated exclusively based on the Foll function do not capture the E2 level well. This data used in the models [7,23,24] was missing E2 values in the middle of the cycle. Margolskee and Selgrade (2011) [26] track the development of the follicle throughout the slow and fast phases of growth until the transformation into corpus luteum and its regression. They [26] model tissue volume of the specific developmental stages of the follicle growth and development. The existing models refer to so-called follicular mass [25,27] or follicular function [7] which are not directly measured. Wright et al. (2020) [27] track “sensitive follicle mass” as it progresses through the biological phases of the human menstrual cycle. They do not allow for estimation of E2 in real-time in field conditions. All these above mentioned models are systems of ordinary differential equations, modelling many hormones as well as structures (CL, follicles). Therefore, there is a need for a mathematical model allowing the understanding of the E2 production by different developmental classes (sizes) of the follicles as well as quantifying the E2 production by each separate follicle (either atretic or healthy). The proposed model can lead to assessment of the E2 production in vivo in field conditions as ultrasonography is a noninvasive method of ovarian structure detection. The calculations of P4 based on the CL size (as described in [7]) together with obtained estimates of IGF1 level based on the P4 concentration (as proposed here) eliminate the need for laboratory testing. The [7] model for P4, combined with ultrasonagraphy results can be used to obtain a crude estimate of the E2 level as well as P4 and IGF1 levels. This field estimation of hormone levels may facilitate the determination of whether a follicular wave is ovulatory or non-ovulatory, which has important commercial implications.

This preliminary model aims to describe (more accurately than previously) the production of estradiol by the ovarian follicles. This is a preliminary model, with parameters estimated based on data from only one cycle of one cow (on every day of the cycle), and data for IGF1 was not gathered in the middle of the cycle and was simulated for these missing days. To improve this model and permit its use in field conditions, data from multiple cycles from multiple cows should be studied, including IGF1 data.

The remaining Sections of the paper are structured as follows. The Results (Section 2) develops and presents a geometric E2 model of the granulosa layer of a follicle (which is responsible for E2 production). This new model for estradiol production is based on the granulosa layer volume of different classes of follicles (and whether or not the follicles are atretic). It uses the follicle size and the developmental stage of the follicles to estimate E2 production. The model also describes the impact of IGF1 and P4 on the level of E2 produced. This is followed by parameter identification (Section 2.6) and model reduction, as well as uncertainty analysis (Section 2.7) and sensitivity analysis (Section 2.8). The existing in vivo model from Boer et al. (2013) of estradiol production is based on training data which was missing E2 values in the middle of the cycle. This was overcome (in the proposed model in Section 2.5) by using the sizes of the follicles to calculate the granulosa volumes and hence obtain the E2 levels.

Additional experimentation was performed and the results from this data were used to optimise the parameters of the geometric model. The experiments are presented in Materials and Methods (Section 4) and the detailed experimental procedure is in the Appendix A. The Conclusions of this study are presented in Section 5.

## 2. Results

We present a model for estradiol production during the bovine estrous cycle which has several improvements over existing models in the literature. The model is based on the sizes of follicles present on the ovary which can be determined non-invasively via veterinary ultrasound. The model comprises the following enhancements:

1. The sizes of the follicles are used to estimate the **granulosa volume** which we propose as a more accurate indicator of the E2-producing capacity of the follicle than a basic sum of follicle diameters which has previously been proposed in [24].
2. The model allows for **lower E2 production rates from atretic follicles** which has been hypothesised in [25,28]. According to McNatty et al. (1981) [28] granulosa cells from the atretic follicles remain steroidogenically active: although they do not have much capacity to produce estradiol they continue to synthesize androgens. Granulosa cells from healthy follicles produce approximately 5 to 100 times more estradiol than granulosa cells from atretic follicles and they produce more estradiol than androstenedione (the latter is produced mainly in theca tissue) [28]. It is shown via model fitting to in vivo data that this hypothesis is supported by the model.
3. The model incorporates the **negative correlation between P4 and E2 production**, especially during the ovulatory wave. This negative correlation between P4 and E2 was also shown in the granulosa cells from the largest follicle by Lee et al. (1989), who found that E2 suppresses P4 [29] in a dose-dependent manner.
4. The model also incorporates the **promoting effect of IGF1 on E2**. IGF1 influences the hypothalamus and pituitary gland which release FSH and LH, which promote E2 production. IGF1 has an impact on the hypothalamus and pituitary gland function [30]. Adam et al. (1998) [31] found that an increase in the IGF1 level stimulated the LH secretion in sheep. Kanametsu et al. (1991) reported that in vitro IGF1 increased production of LH and FSH by the cells in a dose-dependent manner.
5. The data used in the models [7,23,24] was missing E2 values in the middle of the cycle. The same data was used here, but these **missing E2 values were simulated** based on the granulosa volume present at a given time.

The model proposed here is given by the following differential equation:

(1) $$\frac{dE2}{dt}=\left(e_{1}ReF+e_{2}SeF+e_{3}DmF+e_{4}\sum_{i=1}^{N}AtrDmF_{i}\delta \left(t\right)e^{-m(t-{\mathrm{delay}}_{i})}\right)\frac{IGF1^{n_{I}}}{T_{I}^{n_{I}}+IGF1^{n_{I}}}+a_{3}DmF\frac{T^{n}}{T^{n}+P4^{n}}-\alpha_{E2}E2$$

The following follicle classes were used: ReF stands for the sum of granulosa volumes for follicles <5 mm; SeF stands for the sum of granulosa volumes for follicles of 5–8 mm in diameter; DmF stands for the sum of granulosa volumes for follicles of >8 mm in diameter; and $AtrDmF_{i}$ stands for the granulosa volume for atretic dominant follicle *i*. The terms ReF,SeF,DmF and AtrDmF used in Equations (1) and (4) refer to the total volumes of the granulosa layers of these classes of follicles. The terms $e_{i}$ describe the production of E2 by granulosa volume originated from a given class of the follicle, stimulated by IGF1. The terms $a_{i}$ describe the production of E2 by granulosa volume originated from a given class of the follicle, affected by the inhibitory effect of progesterone. The term $\alpha_{E2}$ stands for the clearance rate of E2. The delay is the time lag between the start of the calculations and the first day when a given DmF becomes atretic (AtrDmF). The parameters *m* and $m_{2}$ describe the exponential decrease of the E2 production (both: IGF1 and P4 dependent) by atretic dominant follicles.

The model for IGF1 (described in detail in Section 2.2) is given by

(2) $$IGF1=18.311P4^{5}-59.062P4^{4}+68.983P4^{3}-34.283P4^{2}+5.9711P4+0.6839$$

and the model for granulosa layer volumes (ReF,SeF,DmF and $AtrDmF_{i}$) found in Section 2.1.1) is

(3) $$\mathrm{Granulosa}\mathrm{Volume}=0.1741{\left(\mathrm{follicle}\mathrm{diameter}\right)}^{2}-0.1407\left(\mathrm{follicle}\mathrm{diameter}\right)+0.1288$$

**Figure 1:**
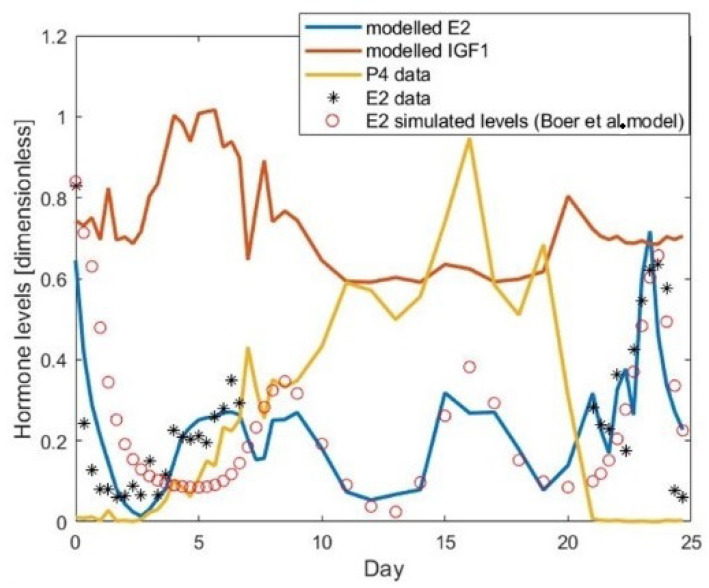
Chosen hormones during the estrous cycle.

Figure 1 shows a comparison between raw E2 and P4 data, the Boer et al. (2017) model of E2 output, and our proposed model of E2 and IGF1. This Figure shows that our proposed E2 model is a better fit to the experimental data (sum of squared errors = SSE = 0.3815) than Boer’s model (SSE = 1.1780), especially at the start of the cycle.

**Figure 2:**
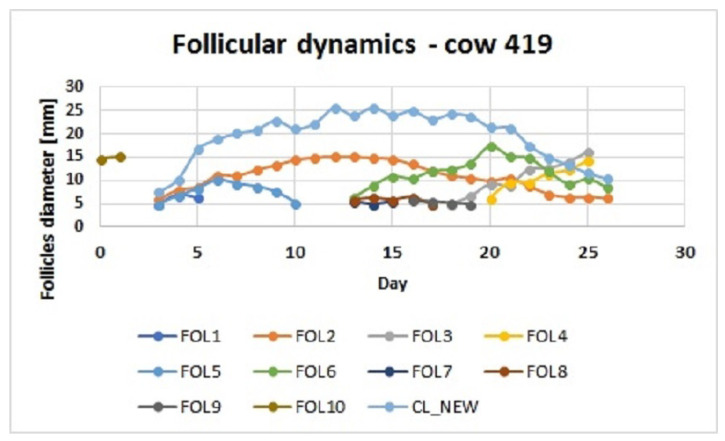
Follicular dynamics.

Figure 2 shows the emergence of two follicular waves for a typical bovine estrous cycle. The first wave in Figure emerges on Day 3 and the second wave emerges on Day 13. The CL is also shown. Most follicles regress, but Follicles 3 and 4 both ovulate (giving a double ovulation).

The drop in the modelled E2 level recorded on Day 7 (in blue in Figure 1) coincides with the P4 peak occurring at the same time. The experimental data for E2 were not available on that day. This drop in E2 is surprising as Figure 2 shows that on Day 6 there is a deviation. This modelled drop in E2 production is because in Equation (1) the production by the atretic secondary follicle (Fol 1) is not modelled on Day 7 (as shown in Figure 2) (however the volume of granulosa of the secondary follicle was added as the SeF class on Day 6). Therefore, this drop in E2 is most likely caused by this simplification.

In the following Sections we present the biological basis and the mathematical and computational development of each aspect of the model, followed by analysis of the parameter uncertainty and model prediction uncertainty.

### 2.1. Modelling of E2 Production

#### 2.1.1. Modelling of Granulosa Volume

The three basic premises of this Section are:

1. Estradiol is produced by granulosa cells, hence the number of granulosa cells determines the E2 production capacity of a follicle. The volume of the granulosa layer is a direct consequence of the number of granulosa cells. The number of granulosa cells per unit volume of granulosa is analysed by histology.
2. We present a quadratic relationship between the diameter of a follicle and its granulosa volume, this allows for estimation of granulosa volume (and hence E2 production capacity of the follicle) from an ultrasound scan of a follicle. This relationship is derived from histology of 98 bovine ovarian follicles. Findings on granulosa thickness are consistent with the literature. A protocol for determining granulosa volume from a histology image is presented in the Appendix A. The method is based on a geometrical model of the granulosa as an axisymmetric three dimensional ellipsoid.
3. Jaswant and Adams [32] noted a decrease in follicle wall thickness in dominant follicles in the late static and regressing stages of the cycle compared to dominant follicles in growing and early static stages. It is also stated in the same paper that subordinate follicles have a thinner granulosa layer than dominant follicles of the same size (and that this explains why dominant follicles produce more E2). These differences are noticeable by 3 days after wave emergence. However, the presented model for granulosa volume depends on follicle size only and ignores the stage of the cycle and also whether the follicle is dominant or not. This is because when doing the histology it was not known what stage of the cycle each follicle and whether or not it had been dominant or subordinate - hence there was insufficient data to allow any knowledge of follicle stage to be taken into account in the model.

**Figure 3:**
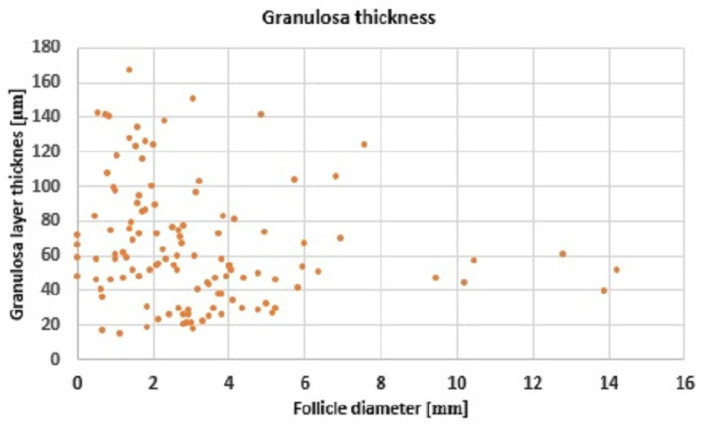
Granulosa thickness at different diameters of the follicle.

The bovine follicle is a complex structure, composed of theca and granulosa layers and the antrum filled with follicular fluid and ovum. We propose a model for the volume of the granulosa cells (which are responsible for producing estradiol) based on the diameter of the follicle. Histology of the follicles (described in Section 4.1) was used to determine the diameters of the follicle and antrum, and the thickness of the granulosa layer in order to create a geometrical model of the follicles. We have used an idea similar to that of Bächler et al. (2014) [33] where a three-dimensional axisymmetric follicle model was used to calculate the thickness of the granulosa layer in a human follicle, knowing that the average volume of a human ovarian granulosa cell has been determined as 1140 μm${}^{3}$ [34]. In this work, we use histology measurements of the thickness of the granulosa layer in bovine follicles to calculate the corresponding granulosa volume. The protocol for performing the histology and granulosa thickness measurements is presented in the Appendix A. Van Wezel et al. [35] found bovine follicle granulosa layer thicknesses ($r_{G}$) at later stages of follicular development ≈ 50 μm, whereas for bovine small antral follicles $r_{G}$ ranges from 40 to 100 μm. Similar values were reported by Jaswant and Adams [32] which give $r_{G}$ values of 50–65 μm in dominant bovine follicles. However in small bovine follicles (less than 5 mm) Iber and de Geyter (2013) [36] calculated granulosa thickness as being equal to 12.5 μm. The granulosa layer does not exceed a certain thickness [32], due to a lack of blood vessels and the need for supplementation of nutrients from the neighbouring theca layer via diffusion [36]. This maximum granulosa layer thickness is approximately 170 μm in Figure 3, which shows the granulosa thicknesses at different diameters of the follicle obtained in this research. The range of granulosa thicknesses observed largely concur with those of [35] although some follicles had thicknesses greater or lower than those observed in [35]. The larger follicles had granulosa thicknesses in the range 40 to 65 mm, similar to the observations for dominant follicles in [32].

In this section, we propose a model of granulosa volume based on follicle size alone, and deal with differences in E2 production due to dominance/subordinance in Section 2.1.2.

**Figure 4:**
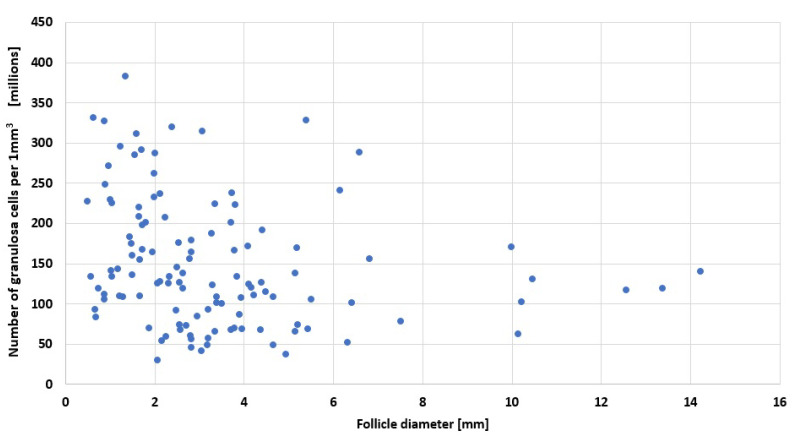
Cell number per volume of granulosa layer.

In addition to measuring granulosa layer thickness (shown in Figure 3) we used microscopy images to calculate the number of cells within a defined volume. The thickness of the histological sections was 5 μm. The results are shown in Figure 4. While there is a spread in the cell density values this has no particular correlation with follicle size. The average number of cells per 1 mm${}^{3}$ of granulosa is 2,320,445.

**Figure 5:**
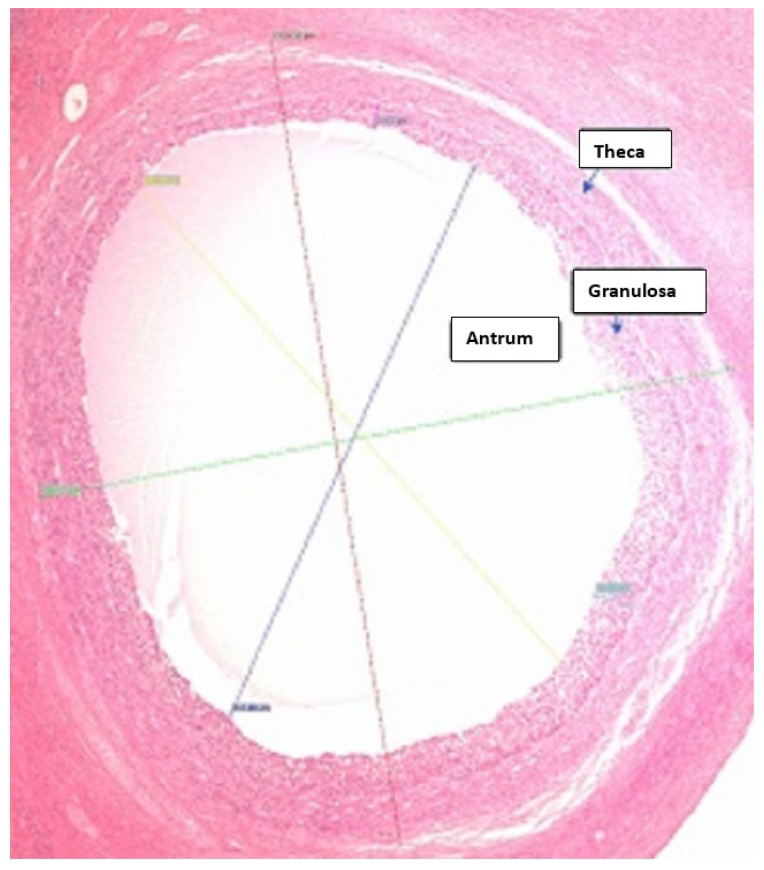
Follicle layers (dimensions 1.93375 mm × 2.27328 mm).

We present a geometrical model for the volume of the granulosa layer, based on the thicknesses of the antrum layer and granulosa layer as follows. Let V= volume of follicle, $r_{A}=$ radius of the antrum with oocyte, $r_{G}=$ thickness of the granulosa layer, and $r_{T}=$ thickness of the theca layer. The follicle layers are shown in Figure 5.

Define $r_{F}$ to be $r_{F}=r_{A}+r_{G}+r_{T}$, the radius of the follicle. $r_{G}=r_{F}-r_{A}-r_{T}$. Assuming the follicle is spherical, the volume of the granulosa layer is $V_{G}=\frac{4}{3}\pi \left({\left(r_{G}+r_{A}\right)}^{3}-r_{A}^{3}\right)$.

However,

$${\left(r_{A}+r_{G}\right)}^{3}=r_{A}^{3}+r_{G}^{3}+3r_{A}r_{G}^{2}+3r_{A}^{2}r_{G}$$

so

$$V_{G}=\frac{4}{3}\pi \left(r_{G}^{3}+3r_{A}r_{G}^{2}+3r_{A}^{2}r_{G}\right)=\frac{4}{3}\pi r_{G}\left(r_{G}^{2}+3r_{A}r_{G}+3r_{A}^{2}\right)\;\;\;\;\;\;\;\;\;\;\;\;\;\;\;\;\;\;\;\;\;\;\;\;\;\;\;\;\;\;\;\;\;\;\;\;\;\;\;\;\;\;‡$$

Hence $V_{G}$ is a quadratic function of $r_{A}$ once the thickness of the granulosa layer becomes constant.

In the histology experiment, the maximum and minimum of the follicle diameters in a plane section were measured. The results of these showed that follicles were rather ellipsoidal in shape rather than spherical. For this reason calculations were done for an ellipsoid and then measurements of diameter were recalculated for a spherical follicle (since ultrasonography results provide only a two-dimensional image of the follicle). Assuming oval-shaped cross-sections of the follicle, the formula for the volume of an ellipsoid was used.

**Figure 6:**
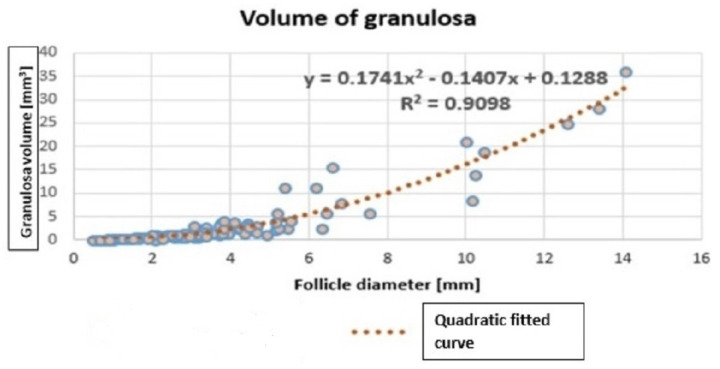
The model of volume of granulosa.

Equation (‡) could be used to estimate the volumes of granulosa for the 98 follicles. However, as only two dimensions were measured, we assumed that the third dimension was the average of the two recorded dimensions (i.e., $r_{A3}=\frac{r_{A1}+r_{A2}}{2}$ and $r_{G3}=\frac{r_{G1}+r_{G2}}{2}$). This produced an ellipsoidal function for granulosa volume (not shown here), which was used on the vertical axis of Figure 6. The granulosa volumes are graphed in Figure 6 showing the follicle diameter on the horizontal axis. A quadratic function of follicle diameter was found (using least squares regression) to estimate granulosa volume, based on Figure 6. This use of a quadratic is motivated by the quadratic function in Equation (‡).

Hence the volume of the granulosa layer is approximated by

$$V_{G}=0.1741x^{2}-0.1407x+0.1288\;\;\;\;\;\;\;\;\;\;\;\;\;\;\;\;\;\;\;\;\;\;\;\;\;\;\;\;\;\;\;\;\;\;\;\;\;\;\;\;\;\;\;\;\;\;\;\;\;\;\;\;\;\;\;\;\;\;\;\;\;\;\;\;\;\;\;\;\;\;\;\;\;\;\;\;\;\;\;\;\;\left(3\right)$$

where *x* is the diameter of the follicle. Equation (3) is derived from histology of 98 bovine ovarian follicles. Equation (3) is later used to estimate the E2 produced by each follicle.

The model is a good fit for follicles below 5.5 mm and above 10.5 mm. The spread in values for follicles in the range 5.5 to 10.5 mm may be due to the differences in granulosa layer thicknesses in dominant and subordinate follicles of similar sizes as observed by [32]. This spread may also be due to the developmental status of the follicle (healthy or atretic). Unfortunately, the number of larger follicles is decreasing throughout the follicular wave, so the number of larger follicles was less than the number of small follicles present on the ovary. Note that in the second, third or even fourth follicular wave there is always present an atretic dominant follicle from the previous follicular wave at a new wave emergence (as demonstrated in Figure 2).

#### 2.1.2. Role of Follicle Size and Developmental Stage on E2 Production

In the previous subsection we outline a model for granulosa volume based on the diameter of a follicle—which is measurable by ultrasound. However, it is known that granulosa volume also depends on the developmental stage of the follicle and on whether it is a dominant or subordinate follicle. Further, the E2 production from granulosa cells differs between dominant and subordinate follicles when the dominant follicle moves to the LH-dependent phase of growth. In this section, we elaborate on these factors and explain how these effects are captured in our model of E2 production.

The granulosa and theca layers combined are referred to as the follicle wall. Jaswant and Adams [32] have noted a significant decrease of follicle wall thickness in dominant bovine follicles at late-static and regressing stages compared to growing follicles and dominant follicles on Day 17. They refer to the side of the wall facing the ovarian surface as the exposed side and the side of the wall further from the ovarian surface as the non-exposed side. The difference in dominant wall thicknesses on the exposed and non-exposed sides is due to differences in granulosa layer thicknesses and theca internal thicknesses on the exposed and non-exposed sides.

Furthermore, Ref. [32] explains why the subordinate follicle produces less estradiol than dominant follicles of the same size. They have recorded that subordinate follicles had a thinner granulosa layer. They did not observe thinning of the wall on the exposed side. These changes occurred at the time of selection. That difference in granulosa thickness between dominant (10–11 mm in diameter) and subordinate (5–7 mm in diameter) follicles was noticeable by 3 days after wave emergence. The dominant follicle causes suppression of E2 production in subordinate follicles by suppressing FSH when it moves to the LH-dependent phase of growth. We do not model FSH and LH but we do allow for different E2 production rates between dominant and subordinate follicles. The different production rates between dominant and subordinate follicles are caused by the morphological as well as endocrinological differences between different developmental stages of the follicles, as described in Section 1. Preovulatory dominant follicles have a higher E2 production rate than non-ovulatory dominant follicles. This is due to the fact that P4 drops to near zero in the ovulatory wave and no longer has an inhibitory effect on E2 production. The model (Equation (1)) does not have a specific term for preovulatory follicles, but it does depend on P4. Whilst the E2 production rates for recruited and secondary follicles ($e_{1}$ and $e_{2}$) may be identical, we have modelled them separately in Equation (1) due to a possible difference as identified in [25]. Due to this possible difference ([25]), and the fact that there is a difference in the hormones required for growth in small and big follicles, there is a difference in the ability to produce the estradiol by subordinate and dominant follicles which differ in their wall thickness [32]. Atretic follicles have lower E2 production rates than healthy ones (due to degeneration of cell density in the granulosa layer). We propose an exponential decay in the number of active granulosa cells over time. To our knowledge, this is the first time that this decline in the production of E2 by atretic follicles is modelled or quantified. A follicle is regarded as atretic when it becomes smaller than 90% of its maximum diameter.

At high P4 levels, the dominant follicle undergoes atresia and its functional dominance is lost between days 7 and 9 of the estrous cycle, preceded by a decline in E2 secretion of the dominant follicle of the first follicular wave [21].

Granulosa cells from healthy follicles produce approximately 5 to 100 times more E2 than granulosa cells from atretic follicles [28]. Our proposed model (Equation (1)) of E2 production at different follicular developmental stages assumes that the granulosa layer steadily degenerates and results in a smaller quantity of active granulosa cells per unit volume of granulosa layer.

Antral atresia is characterized by fragmentation of the granulosa layer close to the antrum, whereas the basal cells remain intact. In this research, numerous pyknotic nuclei were present in some of the most antral cells of the granulosa layer and in the antrum close to the granulosa layer. A basal atretic follicle is characterized by initial destruction of the most basal granulosa cells (those closest to the theca layer), whereas the granulosa cells closest to the antrum remain connected with each other and are healthy. In the research of Irving-Rodgers et al. [37] basal atresia occurred only in small (<5 mm diameter) follicles. The classification in [37] of atretic follicles is based on the E2:P4 ratio in the follicular fluid. This is difficult to establish in vivo. For this reason, only the histological sections with defragmented/invisible granulosa layers and a large number of pyknotic nuclei were disqualified and these data were not used in creating the geometric model of the follicle. Data from histological slides of four follicles were excluded from the further analysis. Hence, we capture differences in the granulosa layers of atretic dominant follicles by allowing for a different E2 production rate from this class of follicles, rather than via the geometric model of Equation (3). In this work, the follicular dynamics were recorded for development of follicles from approximately 5 mm in diameter to the ovulatory size.

### 2.2. Model of IGF1 Concentrations

Unfortunately, although the proposed model for E2 production (Equation (4)) includes the effect of IGF1 on promoting E2 production, raw data for IGF1 was not available. Note that in [38], a regression model for IGF1 was built based on DIM (day in milk). This model could have been used here, but again the data was not available to do so. To overcome this, a model for IGF1 based on P4 levels was created. This is motivated by three factors. Firstly, the relationship between P4 and IGF1 was noted by Lagendijk et al. (2008) [39] which shows that there exists a positive relationship between IGF1 and P4 levels in vivo during the early luteal phase. Secondly, in unpublished work we have noted clustering of the IGF1 receptor with HSD3B (in dendrograms created for genes involved in the bovine ovarian steroidogenesis). HSD3B converts pregnenolone into P4, which suggests that the IGF1 receptor and HSD3B may be involved in the regulation of the same process. This suggests that IGF1 (through its receptor and HSD3B) plays a role in production of P4. Thirdly, Kawashima et al. in [18] state that “ovarian steroids, rather than the nutrient status, may be related to the cyclic changes in IGF1 secretion from the liver”.

Scaled values for IGF1 and P4 from [18] were used (actual value divided by maximum value). The IGF1 level is highest at low levels of P4 and declines to a steady level after P4 increases. This gives an estimation of the physiological level of IGF1 at a given time of the cycle, and is used in Equation (4).

A polynomial of degree 5 was used to create a model for IGF1 based on P4. This polynomial was then used in the proposed differential equation for E2 in Section 2.4. Note that the peak of IGF1 concentration occurs at the start of the estrous cycle. Thus, the peak of IGF1 coincides with the low levels of P4 of growing corpus luteum (CL) origin. This fact is not depicted by the proposed model for IGF1, which does not distinguish between the low concentrations of P4 of early-luteal and late-luteal origin (either growing or regressing corpus luteum). In spite of not distinguishing between early and late luteal phases, the proposed IGF1 model mimics the behaviour of the IGF1 levels during the estrous cycle correctly (when the raw data of P4 levels were used for the validation). Unfortunately, published data of this kind was not available for larger sample groups, so validation of the model was not possible.

To validate the modelled output of IGF1 we have compared the model IGF1 concentration for cow 419 (and others) with the curve of IGF1 levels during estrous cycles in dairy cows [18] and the behaviour of the curve was consistent with experimental results [18].

**Figure 7:**
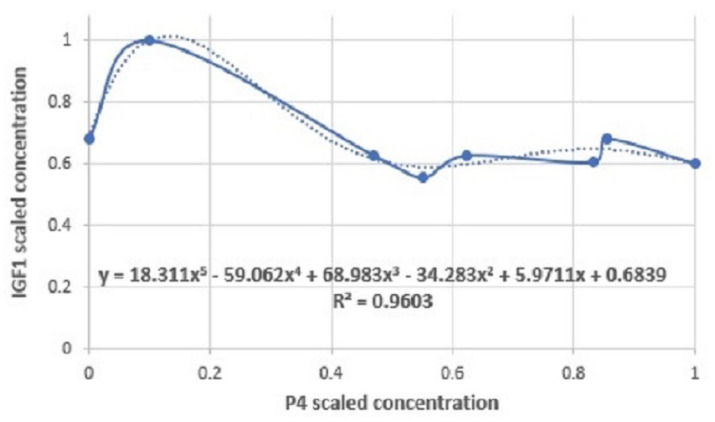
IGF1 as a function of P4.

Data used to build the IGF1 model was collected during normal estrous cycles in n=13 Holstein-Fresian dairy cows [18]. This raw data was read from graphs using GetData Graph Digitizer and results are shown in Figure 7. These data were used to build the polynomial model for IGF1.

Hence the IGF1 value is approximated by

$$IGF1=18.311P4^{5}-59.062P4^{4}+68.983P4^{3}-34.283P4^{2}+5.9711P4+0.6839\;\;\;\;\;\;\;\;\;\;\;\;\;\;\left(2\right)$$

### 2.3. Promotion of E2 Production by IGF1 and Inhibition of E2 by P4

Insulin-like growth factor 1 (IGF1) is one of the factors which stimulates the production of estradiol by granulosa cells [4,40]. A positive Hill function of IGF1 is used in the proposed model of E2 production by granulosa cells to describe this effect. A negative Hill function of P4 is introduced to describe the higher production of E2 at reduced levels of P4 (which happens in the follicular phase). $H^{+}\left(IGF1,T_{I},n_{I},n_{t}\right)=\frac{IGF1^{n_{I}}}{T_{I}^{n_{t}}+IGF1^{n_{I}}}$ is a positive Hill function modelling the stimulatory effect of IGF1 on E2 production and $H^{-}\left(P4,T,n\right)=\frac{T^{n}}{T^{n}+P4^{n}}$ models the inhibitory effect of high P4 levels on E2 production. During an ovulatory wave there is a surge of luteinising hormone (LH), which causes a surge of E2. During the ovulatory wave the P4 concentrations are very low, thus $H^{-}\left(P4,T,n\right)$ models the higher production of E2 during the ovulatory wave. Data for LH was unavailable, but this $H^{-}\left(P4,T,n\right)$ serves as a proxy for LH. This allows calculation of E2 levels in field conditions, without a knowledge of the LH levels, as P4 levels can be calculated based on the CL size recorded via ultrasonography [24]. Furthermore $H^{-}\left(P4,T,n\right)$ has the highest output at the lowest level of P4 which was recorded a day prior to ovulation, followed by a slight increase in the P4 concentration the day after, when the size of the dominant follicle(s) is the largest (has the highest capacity to produce E2 compared to days prior to LH peak). Whereas the IGF1 calculated based on the scaled values of P4 (scale 0 to 1) behaves as expected [18], IGF1 is higher during the start of the first follicular wave and then levels off.

### 2.4. The Dataset

In the following Sections we describe the data set, model reduction (reducing the number of parameters and producing Equation (1) from (4), the parameter identification and uncertainty analysis, sensitivity analysis, prediction uncertainty, and comparison with the Boer model.

The experimental dataset used was the same as that used by Boer et al. (2017) [24]. All cows were synchronised and underwent daily ultrasonography during the subsequent cycle resulting in the data for sizes of corpus luteum and follicles during the entire cycle. The cows’ synchronisation involves manipulation of the reproductive cycle by administration of hormones such as GnRH, PGF2α, P4 (depending on the synchronisation protocol). The synchronisation aims to `reset’ the estrus cycle in the herd resulting in cows having estrus at the same time. This results in a higher chance of successful insemination (getting more cows in calf) and maximises labour efficiency. Blood sampling was carried out at 8 h intervals from day 0 to day 6 of the cycle and from day 15 to ovulation, and once daily from day 7 to day 15. Daily blood samples were analyzed for plasma concentrations of P4 during the entire cycle, and E2 from day 0 to day 6 and from day 15 until ovulation. LH values were not recorded for most of the cycle.

### 2.5. Model Reduction: Implementation of the Improved Mathematical Model of E2 Production throughout the Follicle Lifespan

We have based our proposed model on the interactions between P4, IGF1, E2 and the volumes of different classes of growing follicles: 5–6 mm in diameter (ReF); 6–8 mm in diameter (SeF); dominant >8 mm diameter (DmF); and atretic (Atr) follicles.

The proposed models are Equations (4) and (5), and finally Equation (1).

The preliminary model of E2 production is

(4) $$\begin{array}{c} \frac{dE2}{dt}=\left(e_{1}ReF+e_{2}SeF+e_{3}DmF+\Sigma_{i=1}^{11}e_{i+3}AtrDmF_{i}\right)\frac{IGF1^{n_{I}}}{T_{I}^{n_{I}}+IGF1^{n_{I}}}\\ +\left(a_{1}ReF+a_{2}SeF+a_{3}DmF+\Sigma_{i=1}^{11}a_{i+3}AtrDmF_{i}\right)\frac{T^{n}}{T^{n}+P4^{n}}-\alpha_{E2}E2 \end{array}$$

**Figure 8:**
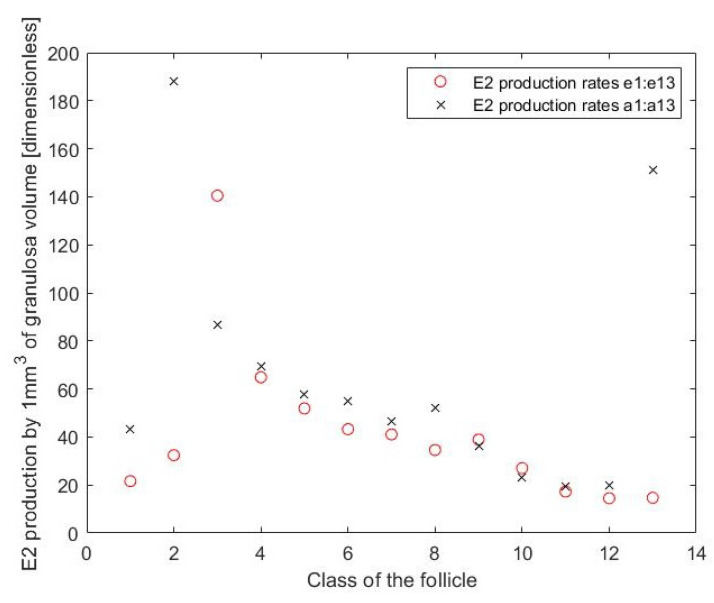
Output of E2 production from Equation (4). “Class of the follicle” refers to terms $e_{1}$ to $e_{13}$ and $a_{1}$ to $a_{13}$. $e_{1}$, $e_{2}$ and $e_{3}$ (class 1 to 3) and $a_{1}$, $a_{2}$ and $a_{3}$ (class 1 to 3) describing the production rate by healthy follicles of ReF, SeF and DmF, respectively. $e_{4}$ to $e_{13}$ (class 4 to 13) and $a_{4}$ to $a_{13}$ (class 4 to 13) stand for declining production rate by atretic follicles throughout their consecutive days of atresia.

This assumes that the different production rates for atretic follicles ($e_{i+3}$) are independent. Here $H^{+}\left(IGF1,T_{I},n_{I},n_{t}\right)=\frac{IGF1^{n_{I}}}{T_{I}^{n_{t}}+IGF1^{n_{I}}}$ is a positive Hill function modelling the stimulatory effect of IGF1 on E2 production and $H^{-}\left(P4,T,n\right)=\frac{T^{n}}{T^{n}+P4^{n}}$ models the inhibitory effect of high progesterone levels on E2 production. The value of $H^{-}\left(P4,T,n\right)$ is high during the ovulatory wave (at low concentration of P4) and low (due to the inhibitory effect of P4 on E2 and Inh production by the follicles) during non-ovulatory follicular waves. The terms $e_{n}$ and $a_{n}$ represent E2 production rates, the terms $e_{1},e_{2}$ and $e_{3}$ and $a_{1},a_{2}$ and $a_{3}$ describe the production rate by healthy follicles of ReF, SeF and DmF class of the follicles, whereas terms $e_{4}$ to $e_{13}$ and $a_{4}$ to $a_{13}$ represent declining production rate by atretic follicles throughout their consecutive days of atresia. Optimising parameters of Equation (4) gives the values of $e_{i}$ and $a_{i}$ which are used in Figure 8.

The results for the production rates by respective classes of the follicles motivated simplification of the model, as shown in the following two models.

In Figure 8, there is a pattern of exponential decay from $e_{3}$ to $e_{13}$, and from $a_{3}$ to $a_{13}$. Replacing these constants with exponential terms gives the next proposed model:

(5) $$\begin{array}{c} \frac{dE2}{dt}=\left(e_{1}ReF+e_{2}SeF+e_{3}DmF+e_{4}\sum_{i=1}^{N}AtrDmF_{i}\delta \left(t\right)e^{-m(t-{\mathrm{delay}}_{i})}\right)\frac{IGF1^{n_{I}}}{T_{I}^{n_{I}}+IGF1^{n_{I}}}+\\ \left(a_{1}ReF+a_{2}SeF+a_{3}DmF+a_{4}\sum_{i=1}^{N}AtrDmF_{i}\delta \left(t\right)e^{-m_{2}\left(t-{\mathrm{delay}}_{i}\right)}\right)\frac{T^{n}}{T^{n}+P4^{n}}-\alpha_{E2}E2\;\;\;\;\;\; \end{array}$$

Here *N* is the number of atretic dominant follicles occurring. $AtrDmF_{i}$ is the volume of the granulosa layer of the *i*th atretic dominant follicle (there may be more than one atretic dominant follicle in a follicular wave). delay${}_{i}$ is the number of days for which the *i*th atretic dominant follicle undergoes atresia.

According to the identified model parameters (given in Table 1) the effect of P4 level on E2 production is very minor for all follicle classes except for dominant follicles.

Removing these $a_{i}$ values gives the final proposed model of estradiol production:

$$\frac{dE2}{dt}=\left(e_{1}ReF+e_{2}SeF+e_{3}DmF+e_{4}\sum_{i=1}^{N}AtrDmF_{i}\delta \left(t\right)e^{-m(t-{\mathrm{delay}}_{i})}\right)\frac{IGF1^{n_{I}}}{T_{I}^{n_{I}}+IGF1^{n_{I}}}+a_{3}DmF\frac{T^{n}}{T^{n}+P4^{n}}-\alpha_{E2}E2\;\;\;\;\;\;\;\;\;\;\;\;\left(1\right)$$

Data for the variables used in the model Equation (1) must be collected. They are: diameter of follicles (>5 mm in diameter), atretic state of follicles (inferred from multiple scans), P4 and IGF1 levels from the blood.

The model for IGF1 found in Section 2.2 is given by

$$IGF1=18.311P4^{5}-59.062P4^{4}+68.983P4^{3}-34.283P4^{2}+5.9711P4+0.6839\;\;\;\;\;\;\;\;\;\;\;\;\;\;\;\;\left(2\right)$$

and the model for granulosa layer volumes (ReF,SeF,DmF and $AtrDmF_{i}$) found in Section 2.1.1 is

$$\mathrm{Granulosa}\mathrm{Volume}=0.1741{\left(\mathrm{follicle}\mathrm{diameter}\right)}^{2}-0.1407\left(\mathrm{follicle}\mathrm{diameter}\right)+0.1288\;\;\;\;\left(3\right)$$

### 2.6. Parameter Identification

The values of the parameters in Equation (1) and their uncertainties were determined using a training data set which consisted of a combination of measured and simulated estradiol concentration values over a complete estrous cycle. Measured E2 concentration values were available for the beginning (days 1–7) and end (days 21–25) of the estrous cycle. For the intermediate days of the cycle, the E2 concentration was estimated using the model of Boer et al. (2012) [23] which was validated by Boer et al. (2017) [24] on the same measured data used here.

The parameter values were estimated using Weighted Nonlinear Least Squares (WNLS) which minimizes a weighted sum of the squares of the E2 level prediction errors. The measured E2 concentrations were given a weighting of 5 while the simulated data points were given a weighting of 1, reflecting the higher level of uncertainty associated with the simulated values. WNLS was implemented with the Matlab command lsqnonlin using the trust-region-reflective algorithm.

The clearance rate and Hill function parameter values are shown in Table 2. The parameter values for the E2 production rates based on follicle sizes for different classes of healthy and atretic follicles are shown in Table 3.

### 2.7. Parameter Uncertainty Analysis

The parameter values from the WNLS step in Section 2.6 were used to form a prior distribution of the parameters (p(θ)). This is combined with the likelihood function ($p(y_{d}|\theta ))$ derived from the training dataset to form the posterior distribution of the parameters (π(θ)) according to the Bayesian framework Equation (6) [41]. The formation of the posterior distribution allows for quantification of the uncertainty of the parameter estimates.

(6) $$\pi \left(\theta \right)=p\left(\theta |y_{d}\right)=\frac{p\left(\theta \right)p\left(y_{d}|\theta \right)}{p(y_{d})}$$

The prior distribution of the parameters was formed as a log-uniform distribution for the logarithm of the parameters over the interval 0.6–1.6 times the logarithm of the parameter values identified by WNLS. The likelihood function is described in Equation (7). The measured E2 data was assumed to contain independent Gaussian noise with a variance $\sigma_{i}^{2}$ at each time point *i* equal to 10% of the measured value. The simulated E2 values in the training set were assumed to contain independent Gaussian noise with a variance $\sigma_{i}^{2}$ at each time point *i* equal to 50% of the measured value.

The likelihood of the measured data $y_{d}$, given the parameters θ, is

(7) $$p\left(y_{d}|\theta \right)=\prod_{i=1}^{n}\frac{1}{\sqrt{2\pi \sigma_{i}^{2}}}\exp\left(-\frac{{\left(y_{i}\left(\theta \right)-y_{d,i}\right)}^{2}}{2\sigma_{i}^{2}}\right)$$

Here $y_{i}\left(\theta \right)$ is the model output at time $t_{i}$ given θ.

Markov-Chain Monte-Carlo (MCMC) sampling was applied to generate 1000 draws from the posterior distribution from which a 95% credible region for the parameters is derived. The method for forming the log-posterior and the MCMC samples and the Matlab code for implementation is given in [41], where inserting Equation (7) into Equation (6) and taking the logarithm gives the log-posterior.

**Figure 9:**
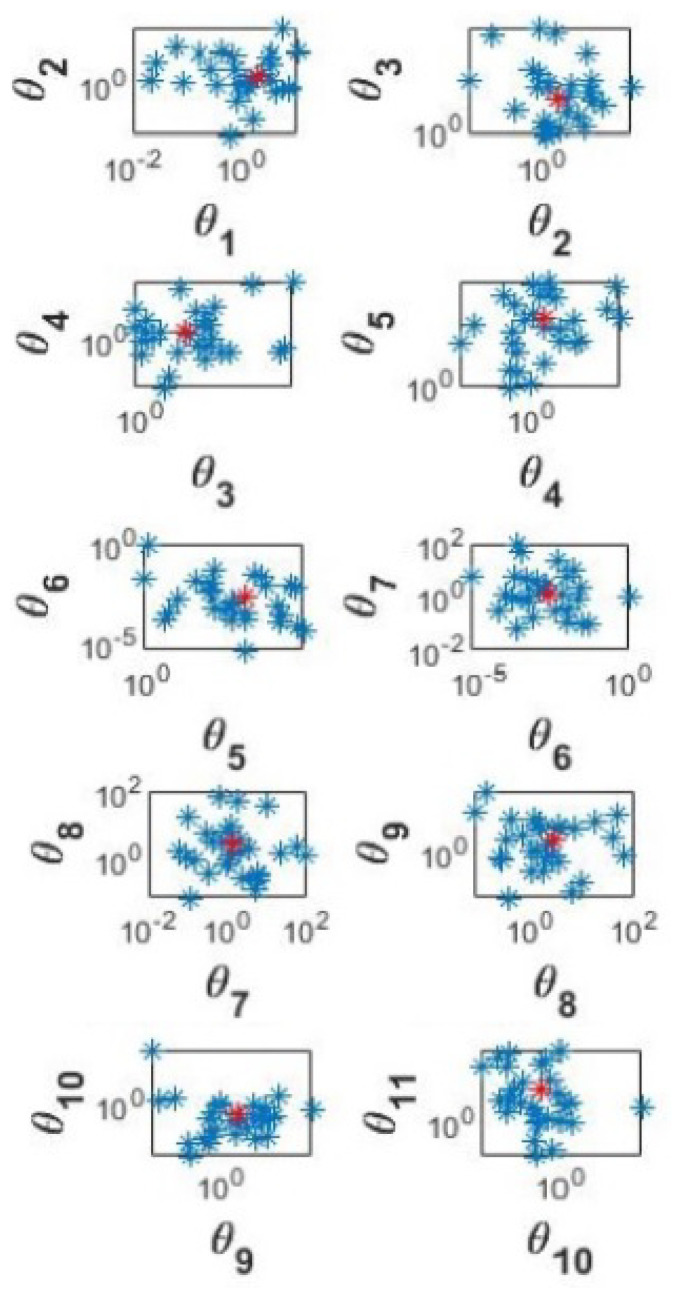
90% confidence regions for the parameters from Equation (1). $\theta_{1}$ to $\theta_{11}$ stand for $\log_{10}$ of the parameters $e_{1}$, $e_{2}$, $e_{3}$, $a_{3}$, n, T, $T_{I}$, $n_{I}$, $\alpha_{E2}$, m and $e_{4}$, respectively. Expected values are shown by the red points.

Figure 9 shows 90% confidence regions for the parameters from Equation (1). For convenience the values are shown on two-dimensional graphs, where parameter $\theta_{i+1}$ is graphed against parameter $\theta_{i}$. The scales on the horizontal and vertical axes reveal the parameters with the largest confidence intervals and greatest uncertainty.

Parameters such as $\theta_{7}$ ($T_{I}$) and $\theta_{8}$ ($n_{I}$) have a high uncertainty. This may be due to the data not revealing very much information of these parameters, possibly caused by use of the simulated data for E2 levels during the middle of the estrous cycle, which was given a high uncertainty in the likelihood function.

### 2.8. Local Sensitivity Analysis

The sensitivity of estradiol predictions to each of the model parameters was investigated with a local sensitivity analysis, i.e., the partial derivatives of E2 concentration with respect to each of the parameters were derived from the solution to Equation (1) with the parameter values as identified above in Table 2 and Table 3. This used a scaled Jacobian matrix calculated using Matlab.

**Figure 10:**
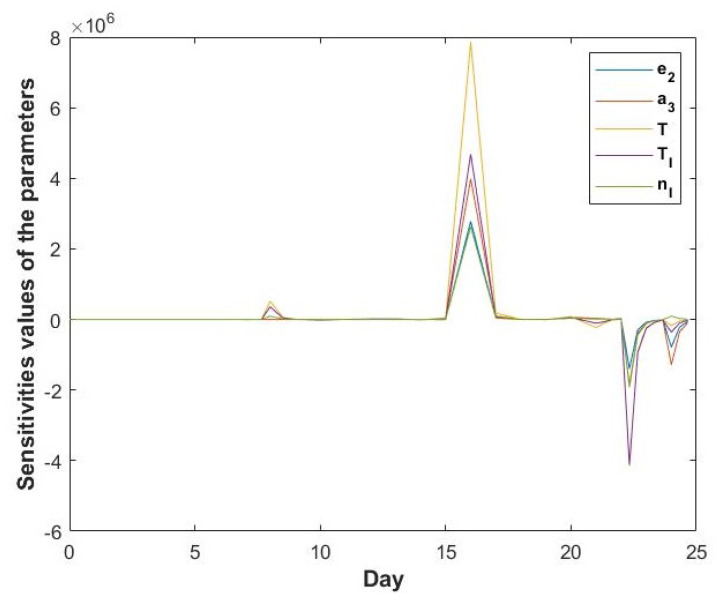
Sensitivities of the parameters in Equation (1).

The results of this sensitivity analysis are shown in Figure 10, which shows the parameters to which E2 is the most sensitive. This shows that the E2 values are most sensitive to the parameters in the middle and the end of the cycle. Therefore small perturbations in the values of parameters have a large effect on the prediction of E2 concentration during the ovulatory wave. The sensitivities of E2 to the parameters in the middle of the cycle, may be due to the availability of only simulated data. The parameters in Equation (1)) to which E2 is most sensitive are $T_{I},T,a_{3},n_{I}$ and $e_{2}$ (listed here in approximately decreasing order of sensitivity). The high sensitivity of the model at the end of the cycle may be due to the declined levels of IGF1 (in the middle and at the end of the cycle) and lower levels of P4. The high sensitivity of the model solution to the parameter values, occurring at the end of the cycle (during the ovulatory wave) might be due to the higher ability to produce E2 by the dominant follicle of the ovulatory wave compared to the dominant follicle of the non-ovulatory waves. However, to avoid over-parameterisation, the dominant follicles of non-ovulatory and ovulatory waves are not distinguished in the model. This is partially addressed in Equation (1) by the impact of low concentration of P4 on the E2 levels recorded during the ovulatory wave.

### 2.9. Prediction Uncertainty

The effect of the parameter uncertainty on the uncertainty of the E2 level predictions was investigated using the method and the code provided in [41]. The 1000 MCMC samples drawn from the posterior distribution of the parameters as described in Section 2.7 were each inserted into the model Equation (1) giving 1000 predictions for the time series of E2. The prediction uncertainty is illustrated by the resulting 90% confidence interval for the predictions in Figure 9.

**Figure 11:**
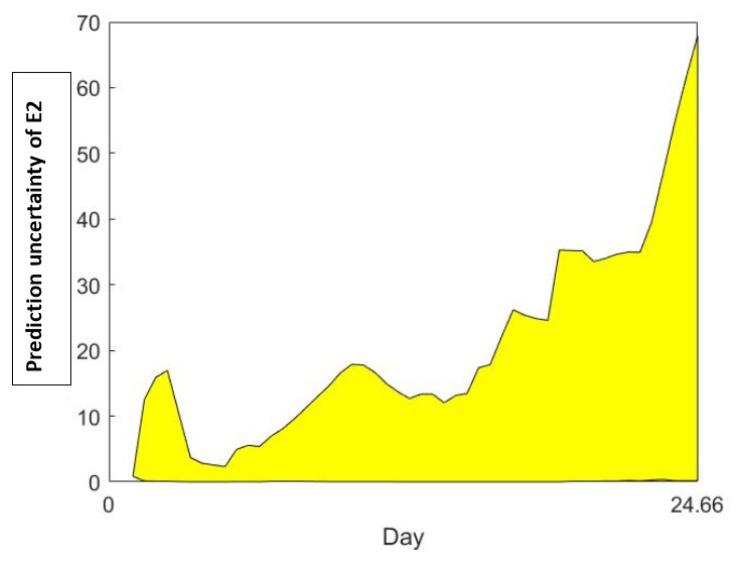
Model E2 Prediction Uncertainty.

The results of the prediction uncertainty analysis are shown in Figure 11, using the technique used in [41].

The parameters with large uncertainty (listed in Figure 9) and high sensitivity (listed in Figure 10), create the most prediction uncertainty. Therefore, the values of these parameters should be estimated for a larger number of cows to capture their variations and to minimise inaccuracy of the ultrasound measurements. In addition, a separate model modelling only E2 levels during either ovulatory or non-ovulatory wave could be constructed for a larger number of animals.

Figure 11 shows the E2 prediction uncertainty for Equation (1). Note that the prediction uncertainty is very high close to the end of the cycle.

### 2.10. Parameter Values and Simulation Results

Values of E2 and P4 were scaled as per [24], which resulted in dimensionless values between 0 and 1. The concentration of IGF1 was simulated using the model of IGF1 described in Section 2.2 using P4 concentration data (scaled).

The maximum volumes of granulosa of each class of the follicle in the dataset are given in Table 4. These values are the scaling factors for a given class. The parameter values from Table 3 were divided by the respective scaling factors (from Table 4) to give Table 5. Applying this scaling gives values between 0 and 1 for the E2 production rates for granulosa volumes in each class.

**Figure 12:**
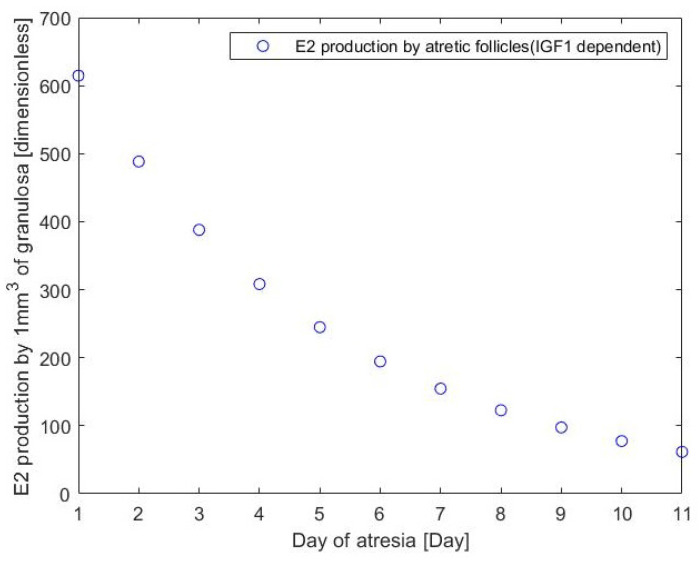
Production rates of E2 by atretic follicles (due to the $e_{4}$ term in Equation (1)).

Figure 12 shows that (based on available data) the IGF1 dependent ability to produce E2 by atretic follicles is large at the start of atresia and declines gradually. The P4 dependent ability to produce E2 by atretic follicles is equal to zero (so no such parameter is included in the model). This is probably due to the shift from E2 to P4 dependent production which occurs in the atretic follicles. In the healthy follicles, the ability to produce E2 increases with the size of the follicle. This difference between production of E2 by smaller and dominant follicles was also recorded by Gutierrez et al. [4].

**Figure 13:**
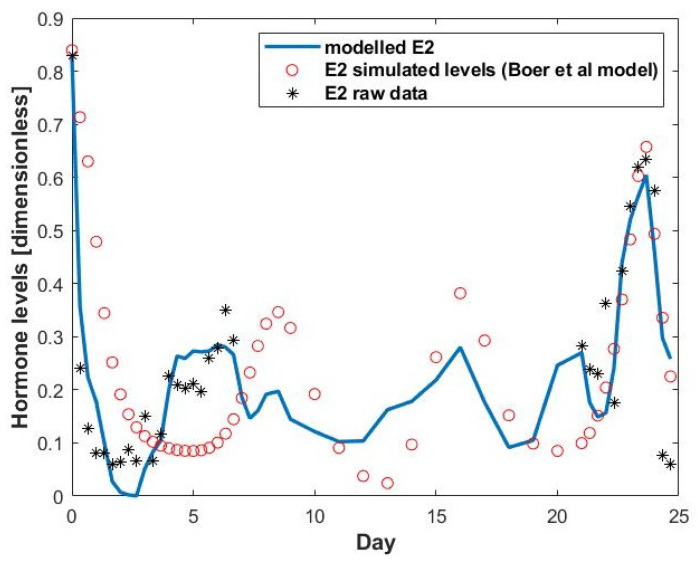
Modelled E2 output. The blue curve represents the modelled E2 from Equation (1). The black points are the raw data and the red points are the modelled E2 values from the model in [24].

Figure 13 shows the results of the simulation using Equation (1), and is discussed in the following section.

## 3. Discussion

To date, there is no model for the production in vivo of estradiol by a follicle throughout its development (as opposed to a model for dominant follicles only). Existing models describe estradiol production as a function of follicle function or mass. However there is a model [23] describing the estradiol production as a function of the size of atretic and healthy dominant follicles. We have created in Equation (3) a model calculating the volume of the granulosa cells (which are responsible for producing estradiol) based on the diameter of the follicle (using the diameters of the antrum, the follicle and the thickness of the granulosa layer). To develop our model (Equation (1)) we have used data of follicle diameters during the estrous cycle and corresponding estradiol levels (data for estradiol levels were recorded at the beginning and end of the cycle). The missing data of estradiol concentration in the middle of the estrous cycle was calculated using the Boer et al. (2012) model, which was validated by Boer et al. (2017) on the same dataset which was used in this paper.

As the follicle grows, there is a mathematical relationship between the diameter of the antrum and the thickness of the combined granulosa and theca layer. This relationship continues even after the thickness of the granulosa layer becomes constant (thickness of the granulosa layer is constant after a certain point due to diffusion limitations, as “estradiol production can rise substantially only by increasing the size of the follicle and thus the surface of the granulosa cell layer” [33]). Equation (1) is the first model to estimate estradiol production based on the granulosa volume (rather than the diameter of the follicle); to distinguish between production of estradiol by healthy and atretic follicles; and model the decline of the estradiol production throughout the progression of the follicle atresia.

The proposed model (Equation (1)) takes this drop in estradiol production into account by introducing atretic classes of follicles in the model. This approach assesses the production of estradiol throughout follicle development and atresia in changing hormonal conditions throughout the estrous cycle. The proposed model gives better estimates of estradiol production throughout the cycle than the existing model of Boer et al.

There are five major differences between the proposed model (Equation (1)) and the model of Boer et al. [23,24]. Firstly, Equation (1) uses granulosa volumes (whereas Boer et al. used follicle diameters). Secondly, Equation (1) distinguishes between healthy and atretic follicles (whereas Boer et al. did not) [23,24]. Thirdly, Pring et al. [25] distinguished between recruited, secondary and dominant follicle classes. This distinction is used in Equation (1), but not in the model of Boer et al. [23,24]. Fourthly, Boer et al. only modelled the estradiol produced by the largest follicles, whereas Equation (1) models all follicles. From Table 5, E2 production from recruited and secondary follicles is small but not insignificant. Fifthly, Boer et al. did not use IGF1 values in the model of estradiol, whereas Equation (1) does.

Figure 13 shows a comparison of E2 values between raw data, the Boer et al. (2017) model, and the proposed model (Equation (1)). This Figure shows that the proposed model is a better fit to the experimental data (SSE = 0.3815) than the Boer et al. model (SSE = 1.1780), especially at the start of the cycle.

Unfortunately, the current model describes the parameters found based on the limited data for a cow with three follicular waves (cow 419) [24]. Thus, the accuracy of the parameter values is limited due to the noise in the data collected using ultrasonography and errors in the concentration of estradiol (measurement error and modelled values for missing data). In addition, there were limitations due to the use of simulated data in the middle of the cycle (rather than observed data). Data from multiple cows could be used to obtain more robust values of the parameters.

Combining the E2 model, the IGF1 model, and the geometry model (Equations (1)–(3)) with a model of progesterone production at different volumes of corpus luteum [23] might result in an improved model estimating the concentration of P4, IGF1 and E2 based on the ultrasound results.

## 4. Materials and Methods

### 4.1. Histology of Bovine Follicles

Histology of the follicles was used to create a geometric model of the follicles, and in particular, to model the volume of the granulosa layers. This was done to create a model allowing calculation of the amount of granulosa and hence the estradiol output based on ultrasonography of the ovaries in vivo. To date there are models of estradiol output during the estrous cycle, but unfortunately they refer to so-called “follicular mass” [25] or “follicular function” [7] which were not directly measured. We have created a model allowing calculations of the volume of the granulosa cells (which produce estradiol) based on the diameter of the follicle. To test our model we have used the data from [24] (and additional Teagasc data for follicle sizes during the cycle) of follicle ultrasonography and hormone concentration in serum.

A protocol for determining granulosa volume from a histology image is presented in Appendix A.

### 4.2. Analysis of Boer et al. 2017 Model of Estradiol Production

There exist several models of hormonal concentrations during the estrous cycle. One of them is the system of Boer et al. 2012 [23] and 2017 [24], where the concentration of estradiol during the bovine estrous cycle is described by the following differential equation:

$$\frac{dE2}{dt}=c_{Foll}^{E2}Foll^{2}-c_{E2}E2\;\;\;\;\;\;\;\;\;\;\;\;\;\;\;\;\;\;\;\;\;\;\;\;\;\;\;\;\;\;\;\;\;\;\;\;\;\;\;\;\;\;\;\;\;\;\;\;\;\;\;\;\;\;\;\;\;\;\;\;\;\;\;\;\;\;\;\;\;\;\;\;\;\;\;\;\;\;\;\;\;\;\;\;\;\;\;\;\;\;\;\;\;\;\;\;\;\;\;\;\;†$$

Here Foll stands for the sum of the sizes of the dominant follicles as described by Boer et al. [24]: “The diameters of the dominant follicles (new and regressing) on each day were summed to give a single curve for the follicle diameter, which was required because follicular endocrine activity in the model is represented by continuous functions for E2 and inhibin (Inh) production”. $c_{E2}$ is the clearance rate of estradiol, $c_{Foll}^{E2}$ describes the rate of the production by the follicles and $\frac{dE2}{dt}$ denotes the rate of change of estradiol.

To obtain a concentration of estradiol during the middle of the cycle we have used the model of Boer et al. (2012), which was validated [24] on the same dataset used to create the model of estradiol presented in this paper. Unfortunately, this dataset has missing data for estradiol, as the E2 level was recorded only from day 0 to day 6 and from day 15 until ovulation.

The values of parameters in † were either known or bounds for these parameters were given in [24]. To obtain approximations for the data on the missing days we have optimised the parameters for the system of ordinary differential equations in [24] (in the cases where only bounds for the parameters were given in [24]). Then this system of differential equations was solved. This optimisation was performed in Matlab version 2015b.

## 5. Conclusions

This model describes the exponential decline of the production of estradiol by the atretic follicles while atresia progresses. This is the first time to our knowledge that this process has been quantitatively described. To date, there was no model of the production in vivo of estradiol by a follicle throughout its development (as opposed to a model for dominant follicles only). Existing models describe estradiol production as a function of follicle function or mass. However there is a model [23] describing the estradiol production as a function of the size of atretic and healthy dominant follicles. This model [23] does not account for the drop in the production of estradiol in atretic follicles. The proposed model (Equation (1)) adjusts for this drop by introducing atretic classes of follicles in the model. This approach allows for the assessment of the production of estradiol throughout follicle development and atresia in changing hormonal conditions throughout the estrous cycle.

In the future this could conceivably help in the assessment of the estradiol levels based on ultrasonography results. This goal might be achievable if the initial estradiol, progesterone and IGF1 levels are known; if the sizes of follicles and corpus luteum are recorded by ultrasound (and we can distinguish between healthy and atretic follicles); and an improved version of Equation (1) is used. Another challenge is to reduce the measurement error and operator inconsistencies for ultrasonography by operator training and instrument recalibration. This future model, even if it does not provide accurate predictions of estradiol levels, may still be useful for predicting the time of ovulation (since the times of maximum estradiol and minimum progesterone can be used to predict the time of ovulation).

## Figures and Tables

**Table 1:** Optimised parameter values for Equation (5) describing the effect of IGF1 and P4 on the production of E2 by 1 mm${}^{3}$ of granulosa cells in different classes of healthy and atretic follicles. These are unscaled values.

Class of Follicle	Effect of IGF1 on E2	Effect of P4 on E2
ReF	$e_{1}=2.1097$	$a_{1}=0.0060$
SeF	$e_{2}=2.4335$	$a_{2}=0.0159$
DmF	$e_{3}=7.0811$	$a_{3}=1.7016$
AtrDmF	$e_{4}=21.0704$	$a_{4}=0.0086$

**Table 2:** Optimised parameter values for Equation (1).

Parameter	Value
*T*	0.0020
*n*	54.5738
$T_{I}$	1.3409
$n_{I}$	2.7371
$\alpha_{E2}$	3.9160
E2(0)	0.8298
m	0.2319

**Table 3:** E2 production by 1 mm${}^{3}$ of granulosa cells in different classes of healthy and atretic follicles for Equation (1). These are unscaled values.

Class of Follicle	Effect of IGF1 on E2	Effect of P4 on E2
ReF	$e_{1}=18.7470$	-
SeF	$e_{2}=39.0060$	-
DmF	$e_{3}=468.1235$	$a_{3}=127.543$
AtrDmF	$e_{4}=619.5579$	-

**Table 4:** Scaling factors.

Parameter	Value [mm${}^{3}$]
ReF	10
SeF	15
DmF	65
AtrDmF	39

**Table 5:** Optimised parameter values for Equation (1) describing the effect of IGF1 and P4 on the production of E2 by 1 mm${}^{3}$ of granulosa cells in different classes of healthy and atretic follicles. These are scaled values (divided by the scaling factors in Table 4).

Class of Follicle	Effect of IGF1 on E2	Effect of P4 on E2
ReF	$e_{1}=1.8747$	-
SeF	$e_{2}=2.6004$	-
DmF	$e_{3}=7.2019$	$a_{3}=1.9622$
AtrDmF	$e_{4}=15.8861$	-

## Data Availability

None of the data were deposited in an official repository.

## Sample Availability

Samples of the histology slides are available from the authors.

## Abbreviations

The following abbreviations are used in this manuscript:

IGF1	Insulin-like growth factor 1
E2	estradiol
P4	progesterone
AtrDmF	atretic dominant follicle
AtreSeF	atretic secondary follicle
ReF	Recruited follicles
SeF	Secondary follicles
DmF	Dominant follicle

## Appendix A. Histology Experimental Procedure

The ovaries of 6 cows (at unknown stages in their cycles) were collected from an abattoir and transported to the laboratory on dry ice. Ovaries were cut in half and transferred into 10% formalin and processed further as per standard methods. Serial sections (5 μm) were cut, deparaffinised in xylene, rehydrated in decreasing concentrations of ethanol and stained with hematoxylin and eosin. Samples were processed according to the standardized procedure developed based on Humason [42]. This consisted of the following steps:

1. Fixation of ovary in 10% formalin (at least 10 times more volume of fixative than volume of tissue) for at least 24 h (follicles were halved for better penetration of fixative buffer).
2. Washing the samples in water for 3 h to remove the formalin.
3. Dehydration: Washing for 1–2 h in graded concentrations of ethanol (50, 70, 90, 95%).
4. Clearing: Washing in 2 changes of xylene for 1–1.5 h.
5. Embed tissue in melted paraffin. After it solidifies, remelt, remove from paraffin and transfer to new liquid paraffin.
6. Cutting into 5 μm sections using microtome.
7. Mounting on glass slides. Sections of the ribbon were spread on hot (2–5 ${}^{\circ }$C lower than melting point of wax) distilled water and then mounted on separate glass slides. One corner of the ribbon was picked up with a pair of forceps. The free end was then placed on the water and the remainder lowered onto the surface. It was stretched slowly to remove creases. The shiny side of the wax was downwards, to ease removing creases. The slides were dried at ambient temperature.
8. Staining: haematoxylin and eosin
  (a) Deparaffinization:
    Xylene 1 for 10 min
    Xylene 2 for 10 min
  (b) Rehydration:
    100% Ethanol (agitation done from time to time) for 2 min
    90% Ethanol (agitation done from time to time) for 2 min
    80% Ethanol (agitation done from time to time) for 2 min
    70% Ethanol (agitation done from time to time) for 2 min
    Bring container with tap water, put slides into that container and put the container under running water for 5 min.
  (c) Staining
    Haematoxylin wash for 6 min
    Bring container with tap water, put slides into container and put container under running water for 4 min.
    Eosin—for 2 min
    60% Ethanol (10 dips, 1 dip per second)
    70% Ethanol (10 dips, 1 dip per second)
    80% Ethanol (10 dips, 1 dip per second)
    90% Ethanol (10 dips, 1 dip per second)
    100% Ethanol (10 dips, 1 dip per second)
9. Clearing slides were blotted and cleaned one by one before putting into Xylene
  Xylene 1 for 10 min
  Xylene 2 for 10 min
10. Mounting: put a drop of mounting agent (DPX) on a slide and drop the coverslip over the sections. Remove any air bubbles. Coverslipping must be completed before the sections dry up.
11. Microscopy using an Olympus BX41 Mason Technology microscope with ColorView camera and ColorView software.
